# Correction: New silicon substituted BiMeVO_*x*_: synthesis and study of structural properties in relation to ionic conductivity

**DOI:** 10.1039/d3ra90021e

**Published:** 2023-03-17

**Authors:** A. Agnaou, W. Mhaira, R. Essalim, M. Zamama, F. Mauvy, M. Alga, A. Ammar

**Affiliations:** a Laboratoire des Sciences des Matériaux et Optimisation des procèdes, Faculté des Sciences-Semlalia, Université Cadi Ayyad Av. My Abdellah B. P. 2390 Marrakech Morocco; b CNRS, Univ. Bordeaux, Bordeaux INP, ICMCB, UMR 5026, 87 F-33600 Pessac France fabrice.mauvy@icmcb.cnrs.fr

## Abstract

Correction for ‘New silicon substituted BiMeVO_*x*_: synthesis and study of structural properties in relation to ionic conductivity’ by A. Agnaou *et al.*, *RSC Adv.*, 2023, **13**, 8015–8024, https://doi.org/10.1039/d3ra00485f.

The authors regret that the name of one of the authors (A. Agnaou) was shown incorrectly in the original article. The corrected author list is as shown above.

The Royal Society of Chemistry apologises for these errors and any consequent inconvenience to authors and readers.

## Supplementary Material

